# Supplementary material for: Content-rich biological network constructed by mining PubMed abstracts
Source: BMC Bioinformatics. 2004 Oct 8;5:147. doi: 10.1186/1471-2105-5-147 (PMC528731; doi:10.1186/1471-2105-5-147)
Supplement: Additional File 2 — The original results of the above study (non-essential files are deleted to keep the file size under the limit set by BMC bioinformatics). [file 1471-2105-5-147-S2.bz2 › chilibotAdditionalFile2/dip20/109ID7589433E448/html/PIK3R1_IGF1R.html]

 


 **PIK3R1** and **IGF1R** 
  
Found 11 abstracts in PubMed, retrieved 11.  
 

 What does Google say? 
 PDF only 
| .edu only 

---

**Interactive relationship** (e.g. stimulation, inhibition, etc)

**Non-interactive relationship** (e.g. studied together, co-existance, homology, etc.)

- Here we investigate the requirement of MAP MAPK and phosphatidylinositol 3 PI3K  [ **PIK3R1** ]  kinases in the transformation of rat intestinal epithelial RIE cells by oncogenic forms of insulin receptor gag IR, insulin like growth factor 1 receptor  [ **IGF1R** ]  gag IGFR, and v Src.  Ref: 11103940 Oncogene, 2000
